# Decline in semen quality among infertile men in Brazil during the past 10 years

**DOI:** 10.1590/S1677-5538.IBJU.2014.0186

**Published:** 2015

**Authors:** Edson Borges, Amanda Souza Setti, Daniela Paes de Almeida Ferreira Braga, Rita de Cassia Savio Figueira, Assumpto Iaconelli

**Affiliations:** 1 Fertility, Centro de Fertilização Assistida, São Paulo, Brasil; 2 Instituto Sapientiae, Centro de Estudos e Pesquisa em Reprodução Humana Assistida, São Paulo, Brasil

**Keywords:** Fertility, Spermatozoa, Sperm Retrieval, Infertility

## Abstract

**Purpose:**

To investigate whether the semen quality of men undergoing conventional semen analysis is deteriorating over time.

**Materials and Methods:**

We analyzed and compared the sperm count, motility and morphology of 2300 semen samples provided by males undergoing conventional seminal analysis, from years 2000 to 2002 and 2010 to 2012. The incidences of severe oligozoospermia and azoospermia over time were also compared.

**Results:**

A total of 764 sperm samples were analyzed in 2000-2002 and 1536 in 20102012. Over time, the mean sperm concentration/ml decreased significantly from 61.7 million in 2000-2002 to 26.7 million in 2010-2012 (R2=11.4%, p<0.001), the total sperm concentration decreased significantly from 183.0 million to 82.8 million (R2=11.3%, p<0.001), and the percentage of normal forms decreased significantly from 4.6% to 2.7% (R2=9.8%, p<0.001). The incidence of severe oligozoospermia significantly increased from 15.7% to 30.3% (OR: 1.09, p<0.001) and the incidence of azoospermia increased from 4.9% to 8.5% (OR: 1.06, p=0.001).

**Conclusions:**

This study demonstrated a significant time-related decline in semen quality of infertile patients. This finding might have implications on fertility and emphasizes the need for further studies addressing subject's life-style in order to find and reduce the causative agents. Future prospective and multicenter studies including representative samples of the general population are needed to confirm whether semen quality is really declining.

## INTRODUCTION

During the past decades several studies have focused on the investigation of seminal quality. A meta-analysis of 61 studies found a significant global decline in the average sperm concentration from 113 to 66 million/ml among men with no history of infertility, between 1938 and 1991 (1). The results of this meta-analysis, showing that sperm density had declined globally by about 50% during the second half of the last century, attracted significant attention and has been a matter of debate. Five years later, a reanalysis of 56 studies confirmed a significant decline in sperm density only in the United States and Europe (2). In an extended meta-analysis of 101 studies, Swan et al. (3) confirmed a decline in sperm density in the period from 1934 to 1996.

Since the publications of Carlson's meta-analysis several laboratories have analyzed their data retrospectively to study trends in their own country and an intense scientific debate was initiated. Despite some studies have suggested that there has been a decline in sperm quality (4–11) others found no significant decline in sperm quality over time (12–19) (reviewed by Jouannet et al. (20) and Merzenich et al. (21)).

A previous Brazilian study retrospectively investigated the quality of donated semen samples and a decline in both sperm count and normal sperm morphology was observed (8). To our knowledge, such an investigation has never been conducted in Brazilian sub fertile couples attending an assisted fertilization center for conventional semen analysis. Therefore, the objective of this study was to investigate if the seminal quality of men undergoing conventional semen analysis is deteriorating over time.

## MATERIALS AND METHODS

### 

#### Experimental design

This retrospective cohort study was performed in a private fertilization center. The sperm count, motility and morphology of 2300 semen samples originating from men undergoing conventional seminal analysis, from years 2000 to 2002 (n=764) and 2010 to 2012 (n=1536) were analyzed. The characteristics from semen samples collected from 2000-2002 were compared to those from samples collected from 2010-2012. The incidences of azoospermia and severe oligozoospermia (sperm concentration <10×10^6^/ml) were compared between the groups.

A written informed consent was obtained, in which patients agreed to share the outcomes of their own exams for research purposes, and the study was approved by the local institutional review board.

#### Semen collection and analysis

All semen samples were collected in the laboratory. After liquefaction for 30 minutes, semen samples were evaluated for sperm count, motility and morphology. The volume of the ejaculate was determined by aspirating the liquefied sample into a graduated disposable pipette. Sperm counting and motility assessment were performed following the instructions of the counting chamber manufacturer (Makler counting chamber, Sefi Medical Instruments, Haifa, Israel). The counting chamber was heated at 37ºC in a heating stage prior to use. The sample was homogenized, by moving gently the container, and a volume of 3-5µL of semen sample was transferred to the center of the chamber. Sperm count was performed in 10 squares of the chamber. The total sperm count is the end concentration expressed as 10^6^ spermatozoa/ml. Sperm motility was assessed in 100 random spermatozoa by characterizing them as (i) grade A (rapid progressive motility), grade B (progressive motility), grade C (non progressive motility) and grade D (immotile) and the motility was expressed as percentage. Sperm morphology was evaluated on air-dried smears, fixed and stained by the quick-stain technique (Diff-Quick; Quick-Panoptic, Amposta, Spain). A total of 200 sperm cells were characterized as morphologically normal or abnormal and the final morphology was expressed as percentage.

### Statistical analysis

Data were expressed as mean±standard deviation (SD) for continuous variables, and percentages were used for categorical variables. Mean values were compared by Student's t parametric test or Mann-Whitney non-parametric test. Percentages were compared by the Chi-squared or Fisher exact test, only when the expected frequency was five or lower. Linear regression analyses, adjusted for male age and period of abstinence, were used to investigate trends over time in sperm count, motility, and normal morphology, giving multiple coefficient of determination (R^2^) for each model. Logistic regression, adjusted for male age and period of abstinence, was used to investigate trends over time in the incidence of severe oligozoospermia and azoospermia, giving odds ratios (ORs), with 95% confidence intervals (CIs), as the effect estimates. A p value of <0.05 was considered statistically significant. Data analysis was conducted using MINITAB 16 Software.

## RESULTS

**Figure 1:**
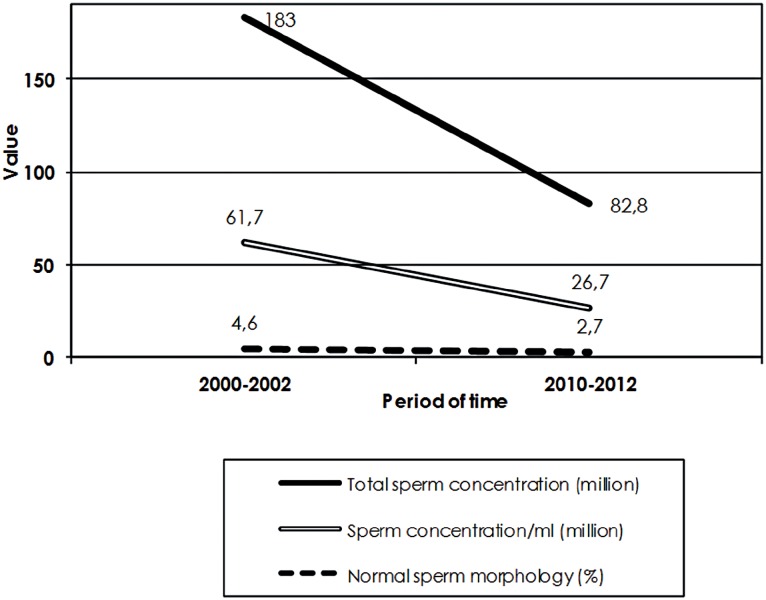
Illustration of differences in semen characteristics.

A total of 764 sperm samples were analyzed in 2000-2002 and 1536 in 2010-2012. Mean male age was 35.7±7.8 years. The general characteristics of sperm samples are shown in Table-1. The comparison of semen sample characteristics between the two groups is shown in Table-2 and Figure-1. Mean male age, days of abstinence and progressive sperm motility were similar between the 2000-2002 and 2010-2012 groups. Over time, the mean sperm concentration/ml decreased significantly from 61.7 million in 2000-2002 to 26.7 million in 2010-2012 (R2=11.4%, p<0.001), the total sperm concentration decreased significantly from 183.0 million to 82.8 million (R2=11.3%, p<0.001), and the percentage of normal forms decreased significantly from 4.6% to 2.7% (R2=9.8%, p<0.001). The incidence of severe oligozoospermia significantly increased from 15.7% to 30.3% (OR: 1.09, p<0.001) and the incidence of azoospermia increased from 4.9% to 8.5% (OR: 1.06, p=0.001) (Table-3).

**Table 1:** General characteristics of analyzed semen samples (n=2300).

Variable	Mean	SD	Min	Max
Male age (y-old)	35.7	7.8	15.0	71.0
Days of abstinence	4.2	2.8	0.0	30.0
Semen sample volume (ml)	3.3	1.7	0.1	11.3
Sperm concentration/ml (million)	38.3	46.7	0.0	540.0
Total sperm concentration (million)	116.0	143.0	0.0	984.0
Progressive sperm motility (%)	36.9	18.9	0.0	84.0
Sperm morphology	3.4	2.9	0.0	16.0

values are mean ± SD, unless otherwise noticed. SD= standard deviation; Min= minimum; Max= maximum.

**Table 2:** Comparison of semen sample characteristics between the groups.

Variable	2000-2002 (n=764)	2010-2012 (n=1536)	p-value
Male age (y-old)	35.0±8.6	35.3±8.1	0.318
Days of abstinence	4.2±3.1	4.2±2.7	0.777
Sperm sample volume (ml)	3.4±1.8	3.3±1.6	0.473
Sperm concentration/ml (million)	61.7±69.4	26.7±27.3	**<0.001**
Total sperm concentration (million)	183.0±197.0	82.8±89.5	**<0.001**
Progressive sperm motility (%)	36.4±18.3	36.5±19.2	0.812
Normal morphology (%)	4.6	2.7	**<0.001**
Incidence of severe oligozoospermia (%)	114/726 (15.7)	426/1405 (30.3)	**<0.001**
Incidence of azoospermia (%)	38/764 (4.9)	131/1536 (8.5)	**0.001**

values are mean±SD, unless otherwise noticed. SD: standard deviation.

**Table 3:** Regression analyses’ results for trends over time in semen quality.

Variable	R2 (%)	p value
Sperm concentration/ml	11.4	<0.001
Total sperm concentration	11.3	<0.001
Normal forms	9.8	<0.001
**Variable**	**OR (CI)**	**p value**
Severe oligozoospermia	1.09 (1.06-1.11)	<0.001
Azoospermia	1.06 (1.02-1.10)	0.001

## DISCUSSION

Data presented in this study suggest that the semen quality of Brazilian sub fertile men seems to be deteriorating over time. Our results showed statistically significant differences in the seminal characteristics of the subjects analysed in the time gap of 10 years, i.e., 2000-2002 and 2010-2012 most notably in the sperm concentration and normal sperm morphology, favouring the period time of 2000-2002. It is important to highlight that during the study period, there were very little changes in the techniques and personnel involved in the analysis of semen. Technicians adhered to strict quality control and the equipment used were the same throughout the entire study period.

Our results support previous reports that the sperm quality in human semen seems to be globally declining. Glina et al. (8) found a decrease in sperm concentration in Brazilian donors samples from 1992 to 2003. This investigation was conducted in the same city as was the present study. Moreover, similar findings concerning sperm concentration have been reported in Spanish (9, 22), Scottish (6, 23), French (11, 24, 25), Norwegian (26, 27), Italian (5, 28, 29), Danish (4, 30), Belgian (31), German (10), Austrian (32), Greek (33), Israeli (34, 35), Tunisian (36) Chinese (37) and Canadian (7) men. Conversely, many studies failed to demonstrate a time-related decline in semen quality (12–19, 38–41).

In the present study we also observed that sperm morphology has changed over time. This is in agreement with previous studies (8, 13, 14, 25, 36). On the other hand, many studies failed to demonstrate such association (22, 32, 42). It has been suggested that this parameter may vary over time depending on the classification criteria adopted and evaluation experience (13). In fact, in some studies the morphology was not investigated due to high inter-observer variation (23, 35).

Recently, a recent systematic review from Cocuzza and Esteves (43), concluded that there is no enough evidence confirming a global decline in semen parameters. Curiously, some studies observed that semen quality has not declined nor remained steady, but slightly increased in recent years (15, 42, 44–47). Nevertheless, follow-up studies are necessary to investigate whether this finding is a real phenomenon or purely random variation. The discrepancy in the results obtained in the studies may be explained by selection criteria of volunteers or other confounding factors, such as the number of subjects included in each study. It is noteworthy that the observed time trend in semen quality might be an artifact, since the methodological variances amongst studies might be time-dependent (21).

The causes of the possible decreasing quality in male reproductive function remain to be elucidated. It has been suggested that the increased frequency of male reproductive abnormalities reflect adverse effects of environmental or lifestyle factors, such as occupational and environmental exposures, medications, and sexually transmitted diseases (48, 49). Indeed, the industrial expansion and demanding agricultural activity of South America, along with repeated disrespect for environmental protection measures, are a risk to human populations health (50). However, a recent systematic review reported that there is no scientific evidence of a causative role for endocrine disruptors in the decline of semen quality (43).

Poor semen quality appears to be a common occurrence that is in agreement with the growing need of assisted reproduction worldwide (21). In the present study we observed an increase in the incidence of severe oligozoospermia, which is in line with the observed decline in sperm count, and azoospermia. It is known that azoospermia is common among the infertile population and it has been suggested that its prevalence is likely to increase in infertility clinics (51). Many patients with azoospermia are thought to have a contributing genetic cause. Therefore, there is a concern regarding the risk of transmission of these abnormalities to offspring (52).

The potential drawbacks of this study are: (i) semen analysis data were retrospectively reviewed; and therefore, (ii) we were unable to collect information on potential confounders, including occupation of the subjects, smoking, food habits and level of stress. Moreover, (iii) the inclusion of potential sub fertile men attending an infertility center might be a selection bias.

As suggested by Olsen and Rachootin (53), a monitoring system could ensure that we have a better understanding of developments over the next years. One of the consequences of a possible decline in sperm quality is the increase of infertile couples (50).

## CONCLUSIONS

This study demonstrated a significant time-related decline in semen quality of infertile patients. This finding might have implications on fertility and emphasizes the need for further studies addressing subject's life-style in order to find and reduce the causative agents. Future prospective and multicenter studies including representative samples of the general population are needed to confirm whether semen quality is really declining.
